# Teaching Cariology in Asia and Arabia

**DOI:** 10.1159/000524168

**Published:** 2022-03-21

**Authors:** Christian H. Splieth, Mohamed Hassan Abudrya, Latha Anandakrishna, Lei Cheng, Khalifa S. Al-Khalifa, Baek-Il Kim, Irina Kuzmina, Ahmad Tarabaih, Anas Salami, Yasmine Elhamouly, Julian Schmoeckel

**Affiliations:** ^a^Department of Preventive and Pediatric Dentistry, University of Greifswald, Greifswald, Germany; ^b^Department of Pedodontics and Preventive Dentistry, Faculty of Dental Sciences, M S Ramaiah University of Applied Sciences, Bengaluru, India; ^c^Department of Operative Dentistry and Endodontics, West China Hospital of Stomatology, Chengdu, China; ^d^Department of Preventive Dental Sciences, College of Dentistry, Imam Abdulrahman Bin Faisal University, Dammam, Saudi Arabia; ^e^Department of Preventive Dentistry & Public Oral Health, Brain Korea 21 FOUR Project, Yonsei University College of Dentistry, Seoul, Republic of Korea; ^f^Department of Preventive Dentistry, Moscow State University of Medicine and Dentistry, Moscow, Russian Federation; ^g^Division of Pediatric Dentistry, Department of Developmental Sciences, Faculty of Dentistry, Beirut Arab University, Beirut, Lebanon; ^h^Pediatric Dentistry Department, Mohammed Bin Rashid University of Medicine and Health Sciences (MBRU), Dubai, United Arab Emirates; ^i^Department of Pediatric Dentistry and Community, Faculty of Dentistry, Pharos University in Alexandria, Alexandria, Egypt

**Keywords:** Cariology, Dental education, Undergraduate curriculum

## Abstract

The European Organisation for Caries Research education platform 2020 had the aim to assess the undergraduate curriculum in cariology in Asian and Arabian countries in order to support structured teaching of cariology in these countries with about almost half of the global population. Representatives of 4 Asian and 4 Arabian countries completed a comprehensive questionnaire on structure of dental education in their country in general and the extent, the content, the responsibilities, structure and standardization regarding cariology in particular. In spite of a wide range from very few universities (Lebanon 3) to larger numbers of dental schools (India 313, China 121, Russia 52) there were similar statements on the list of content for cariology teaching. Often the catalogue was close to the Undergraduate Core Curriculum in Cariology (UCCC) covering most of the 5 domains from basic science to dental public health, but a national curriculum for cariology or dentistry was mostly missing. With various departments being involved, a need of coordination is obvious. Most representatives thought it possible and feasible to teach a standardized curriculum in cariology on the basis of the UCCC. In conclusion, many Arabian and Asian countries have implemented modern, evidence-based curricula in their universities, but an obligatory national curriculum in cariology would be advisable to standardize the quality in teaching.

## Introduction

In 2018, European Organisation for Caries Research (ORCA) decided to shift the focus to Asia and Arabia which unite almost 50% of the global population and to designate the Education Platform 2020 to the teaching in cariology in these regions. Based on this meeting during the ORCA congress 2020, this paper reports the outcome of a structured questionnaire assembled for 4 Asian and 4 Arabian countries, and it develops the perspective to support standardized, evidence-based national curricula in cariology for undergraduate students. Despite the decline of dental caries in many countries, it remains to be the most prevalent oral disease with a high global burden [Carvalho and Schiffner, 2019; Kassebaum et al., 2015; Lagerweij and van Loveren, 2015; Petersson and Bratthall, 1996; Splieth et al., 2019]. Thus, it looks advisable to standardize the different aspects in cariology such as terminology [Machiulskiene et al., 2020], caries management [Paris et al., 2020], or education on a global level. Following this approach, an Undergraduate Core Curriculum in Cariology (UCCC) [Schulte et al., 2011] was developed by the ORCA in cooperation with the Association of Dental Education in Europe (ADEE) to structure and standardize evidence-based and comprehensive teaching in cariology on a global level. Starting at the European level, 171 dental schools which were members of ADEE were invited to complete a questionnaire on the structure of teaching in cariology at their institution and on the willingness to develop a common undergraduate core curriculum in cariology. Due to the high and positive response (89%), ORCA progressed with a steering group to develop five domains from the knowledge base, diagnostics, prevention, and therapy to evidence-based cariology in clinical and public health. This draft was specified in a joint workshop in Berlin/Germany 2010 with 75 invited participants from 24 European countries as well as 3 countries from North and South America in five working groups for the different domains, finalized in a subsequent review and published by the chairs of the five working groups [Schulte et al., 2011]. The 5 domains of the UCCC deal with different aspects of teaching cariology which can be obtained by the correlating titles of each domain.

The UCCC was in line with the trend for standardization in teaching and the development of national curricula and education standards in many fields [Bennett et al., 2020], which started in dentistry about 20 years ago [European Society of Endodontology, 2001; Plasschaert et al., 2005; European Academy of Paediatric Dentistry, 2009]. It also created a cooperation with the implementation of according curricula in Colombia, Canada, Chile, Spain, the USA, and the Caribbean [Martignon et al., 2013; Martignon et al., 2014; Fontana et al., 2016; Tikhonova et al., 2020; Abreu-Placeres et al., 2021; Cortés-Martinicorena et al., 2021].

## Materials and Methods

Representatives of different Asian and Arabian countries were contacted to provide information on the situation in undergraduate education in cariology. University teachers of 4 Asian (China, India, Republic of Korea, Russia) and 4 Arabian countries (Egypt, Saudi-Arabia, Lebanon, the UAE), who are also all authors of the article, consented to answer a comprehensive questionnaire on the structure of the dental education in their country which contained the following items:

- undergraduate teaching in general
- extent and the content regarding cariology in particular
- responsibilities between the different departments
- structure of the curriculum in cariology
- measures of standardization

The representatives filled out a standardized questionnaire form and gave a presentation at the ORCA education platform meeting in 2020. Afterwards, the information was condensed and the items were summarized in the tables seen in the result section and presented descriptively.

## Results

Cariology is taught in the depicted four Asian and four Arabian countries with a wide range of numbers of universities obviously related to the population of the country from 3 (Lebanon) to 313 (India) with relevant differences regarding the proportions of public versus private universities (Table 1). However, only few countries have a national curriculum for dentistry or cariology which is mostly not compulsory. Partially, the domain “Cariology” is clearly described, but it is usually split up in courses taught by different departments (Table 2). For example, in India, the departments oral medicine, oral pathology, pedodontics and preventive dentistry, conservative dentistry, and public health dentistry participate in teaching cariology to cover aspects like the aetiology, pathogenesis, epidemiology, diagnosis, and caries management. Specific “stand-alone” departments for cariology rarely exist. If the denomination exists, they are, e.g., part of a combined department for cariology and endodontics. Consequently, no specifically entitled professors/chairs for cariology are mentioned (Table 2).

Mostly the content taught regarding cariology was close to the UCCC covering most of the 5 domains from basic science to dental public health. Apart from the few countries with a compulsory national curriculum for cariology or dentistry, the other representatives thought that a good chance for a standardized curriculum in cariology on the basis of the UCCC exists − not only in their own university, but also on a national level (Table 3). Table 3 also lists the obstacles to the introduction on a national level. Partially, some items or even complete domains were considered to need modification or to be implemented to improve the undergraduate curriculum in cariology in the specific universities or countries. Overall, there was an optimistic outlook regarding the teaching of cariology according to the UCCC in different countries in Asia and Arabia (Table 3).

## Discussion

To our knowledge, this is the first overview on the teaching of cariology in various Asian and Arabian countries. It includes the largest Asian countries and a wide range of Arabian countries representing about half of the global population. As the representatives have a valid insight in the regulations on teaching dentistry in their countries, the answers regarding the existence of a national curriculum and the content and responsibilities for teaching cariology in their own universities can be considered very accurate.

In most Asian and Arabian countries and universities, there seems to be some variation in teaching cariology, but there is a trend to standardize this into a national curriculum, often based on competencies that graduating dentists should fulfil [Li et al., 2021]. Sometimes accreditation boards are responsible to ensure an adequate quality. Within countries, it could be difficult which body or experts have the authority to define the national curriculum. Thus, the approach to define components of these curricula by the according international scientific associations and societies could be an interesting solution. This process was initiated, e.g., by the European Society of Endodontology [European Society of Endodontology, 2001], and the European Academy of Paediatric Dentistry [European Academy of Paediatric Dentistry, 2009], and also by ORCA as the leading global organisation for caries research [Schulte et al., 2011].

The Core Curriculum in Cariology provides a framework for the items that should be taught in cariology during the undergraduate education in dentistry without being too detailed about the current interpretation of the state of art. As caries follows the same biological concepts globally, the approaches to teaching its underlying biological principles, aetiology and epidemiology, prevention, diagnostics, or treatment should also be comparable. Therefore, curricula based on the UCCC were successfully implemented in various countries such as Chile, Colombia, Spain, the USA, and the Caribbean [Martignon et al., 2013; Fontana et al., 2016; Tikhonova et al., 2020]. Like in the present paper the adaptation went into two directions: universities filled the gaps in their curriculum according to the catalogue of the UCCC while some domains were split up or increased. Especially domain V which covers the principles of evidence-based dentistry and also community/public health dentistry could be divided into separate identities because it is likely that this domain could be taught by different departments.

In conclusion, the UCCC presents a very flexible instrument for teaching cariology in a comprehensive approach from biological and epidemiological determinants to disease management which includes the classical preventive, diagnostic, and therapeutic procedures. This was confirmed for the Asian and Arabian countries in this paper. With increasing globalization and migration, the UCCC offers clear advantages for an (inter)national standardization to achieve comparable dental education in cariology. As it was already developed by experts from around the world, it saves resources to organize this process in every country or even university, as positive examples from several countries show.

## Statement of Ethics

Ethics approval was not required according to the regulations of the Ethics Committee at the University of Greifswald, as this publication uses publicly available data or statements by the authors.

## Conflict of Interest Statement

The authors declare no conflict of interest.

## Funding Sources

No external funding was used. The authors completed the questionnaires and wrote the paper in their personal time, mainly paid by the according university.

## Author Contributions

C.H.S. conceptualized the design of the study and the questionnaire and the drafted the manuscript. The Asian and Arabian author gave the details for their country and revised the manuscript. Results and tables were compiled by J.S. who also drafted the manuscript, while M.H.A. drafted the results and critically revised the manuscript.

## Data Availability Statement

All data generated or analysed during this study are included in this article. Further enquiries can be directed to the corresponding author.

## Figures and Tables

**Table 1:** Presence of a national curriculum in dentistry/cariology in different countries in Asia and Arabia

Country	Dental schools (public/private), *n*	National curriculum dentistry/cariology exists	Compulsory	Cariology clearly described domain
Saudi Arabia (KSA)	28 dental schools (20 public/8 private)	No	No	No, but some schools teach cariology as a “stand-alone” course

UAE	5 dental schools (5 private/semi-government)	No, individual schools follow WHO, USA, and UK curricula	Yes, for teaching cariology, but not as national curriculum	Varies: undergraduate schools have base cariology curriculum on the aetiology, pathology, prevention, and treatment of dental caries in different domains

China	112 colleges/universities (mostly state schools)	Yes	Yes, compulsory courses (base courses, clinical courses, and clinical skills training) + optional courses online (national excellent courses)	Yes, it is mainly included as a subsection in a compulsory course of operative dentistry and endodontics It is also involved in courses like oral histopathology, oral biology, preventive dentistry, and paediatric dentistry

Republic of Korea	11 dental schools (6 public/5 private)	No national curriculum for dentistry/cariology	Yes, dental schools should obtain certification from KIDEE	Yes, usually included in preventive dentistry, paediatric dentistry, and conservative dentistry as a subsection

Lebanon	3 dental schools (2 private/1 public)	No national based dental curriculum Each dental school has its own curriculum	No, each university has the freedom to design the curriculum and the assessments methods	No, it is not defined as a separate domain, but certain aspects of cariology are described under different specialties

Egypt	44 dental schools (20 public, 4 community colleges nonprofit, 20 private)	No national curriculum, but international curricula (USA, UK, etc.) are followed	Yes, cariology is incorporated in compulsory preclinical and clinical undergraduate courses	No, but cariology is covered in courses taught by different departments mainly in terms of aetiology, pathogenesis, diagnosis, prevention and treatment

India	313 dental schools with undergraduate programme (42 public/271 private, 26,949 seats) 259 dental schools with undergraduate and postgraduate programme (6,228 seats)	Yes, however, cariology is handled by departments of oral medicine and radiology, public health dentistry, paediatric and preventive dentistry, conservative dentistry and endodontics, oral pathology, and microbiology	Yes, all dental colleges in India have to comply with DCI regulations in terms of curriculum, delivery and assessment. However, universities have freedom in designing the delivery and assessment	No, but certain aspects of cariology are described under each handling department

Russia	52 dental faculties mostly state dental schools/1 private dental school	Yes, and cariology is a module in a curriculum	Yes, but the national curriculum may contain an elective component, which varies from 8 to 30% of the total program and may be planned by the university	Yes, cariology is rather clearly described and part of the module “dentistry” Cariology is taught at the departments for cariology and endodontics, preventive dentistry, pedodontics, and clinical dentistry

**Table 2:** Teaching of cariology with respect to the UCCC in different countries in Asia and Arabia

Country	Chairs/departments/professors for cariology	Which departments teach cariology?	Domains of UCCC taught at representatives’ school	Domains of UCCC feasible to be taught at representatives’ school
KSA Saudi Arabia	No	Restorative dentistry Preventive dentistry Biomedical sciences	No there are similarities and overlaps	Yes, most domains are taught in some dental schools. The domains I–III are covered in cariology courses while domains IV and V are taught in other courses. Domains of UCCC are feasible to be taught at Saudi Arabian dental schools if properly introduced

UAE	No, some universities have it within the department prevention and growth, others under the basic sciences department	Oral epidemiology and biology, dental public health, dental prevention and paediatric dentistry, operative, and restorative dentistry	No exact domains are being taught, but there are many similarities and overlaps in the curriculum	Yes, the five domains of UCCC are taught in our schools, but under different sections and departments: Domains I and II as basic science in the first 2 years of the program; domains III and IV in the restorative and prevention curriculum Domain V is less covered in most undergraduate schools

China	Yes, but usually combined as department of operative dentistry and endodontics	Oral histopathology, oral biology, operative dentistry and endodontics, preventive dentistry, and paediatric dentistry	The courses and teaching methods currently adopted by our school are similar to the UCCC. There are 17 courses related to UCCC which are taught in Sichuan university	Yes, all five domains of the UCCC could be taught in China

Republic of Korea	No separate chairs/departments/professors of cariology	Departments vary but usually preventive dentistry, paediatric dentistry, and conservative dentistry	All domains are taught by my school	Yes, considered to be extremely likely

Lebanon	No specific professors nor department for cariology	Departments of restorative sciences, biological and diagnostic sciences, and developmental sciences	Most of the UCCC has been taught in a spiral curriculum designed to emphasize and revisit clinical themes and competencies of the 5 domains in greater detail	Yes, all 5 domains of the UCCC could be taught

Egypt	No specific professors nor department for cariology	Oral biology, oral pathology, dental public health and community dentistry, paediatric dentistry, restorative dentistry, and endodontics	Domains I–IV are covered to a great extent by different departments in the undergraduate curriculum Domain V is mainly taught in the postgraduate curriculum not for the undergraduates	Yes, domains I–IV are already taught by different departments, domain V needs to be incorporated in the undergraduate curriculum

India	No, as cariology is not specifically recognised by the dental council of India, there are no entitled professors in cariology	Oral medicine − Domain 2 Oral pathology − Domain 1 Pedodontics and preventive dentistry − Domain 1–5 Conservative dentistry − Domain 1–5 Public health dentistry − Domain 2–5	UCCC has yet to gain popularity in India At Ramaiah university of applied sciences, Bangalore, UCCC has been taught as a joint teaching module in 3rd year in 2018 and 2019 with involvement of all 5 departments	Yes, all 5 domains of the UCCC are already taught at faculty of dental sciences, Ramaiah university of applied sciences, Bangalore, India

Russia	No specific entitled professors in cariology. However, at our university there is a department of cariology and endodontics	Varies but mostly departments for preventive dentistry, cariology and endodontics, pedodontics, clinical dentistry, therapeutic dentistry, and preclinics	Our educational curriculum is not based on the UCCC by itself; however, the major part (principles or ideas) is lined up in main and elective parts	Yes, all or at least the majority of the domains of the UCCC could be taught at our dental faculty

**Table 3:** Chance and obstacles for teaching cariology according to the UCCC in Asian and Arabian countries including suggested modifications

Country	Chance and obstacles to standardize undergrade cariology in country according to UCCC	Items/domain needing modification to improve teaching in cariology
(KSA) Saudi Arabia	Yes, UCCC has an opportunity to be introduced to Saudi Arabian dental schools	There is a need to include all domains to be taught under the cariology course

UAE	Yes, UCCC is applicable and can be introduced to our undergraduate students	All domains perfectly fit the UAE curriculum, except domain III, which needs to be modified to fill the gaps and needs of the UAE community and culture. In addition, domain V needs to be tailored to the country's needs and altered according to the available national evidence and epidemiological data

China	Yes, in recent years, a series of reforms have been carried out in the cultivation of undergraduates of stomatology in China. Teaching models, such as problem-, case-, or team-based learning, etc., are explored	Dental education could be started at an earlier stage of undergraduate education, including clinical clerkship, basic practice in laboratory, etc.

Republic of Korea	Yes, but standardization of the curriculum would take a long time as cariology is currently taught in various departments	The scope of domain I is so broad that it seems necessary to reconstruct it more specifically with background knowledge limited to caries

Lebanon	Yes, there is an opportunity to work on standardizing the curriculum between different universities	Limitations in specific competencies of domains 3 and 4 1) assessment of the needs of risk groups (disabled patients) 2) administering preventive measures more appropriately in clinics 3) assessment and monitoring of treatment outcome over time

Egypt	Yes, there is a good chance, as domains’ outcomes are taught by dental schools in Egypt, but it will require some time to standardize it among different universities	Domain III and V need to be modified with respect to the requirements, preferences, and resources of the Egyptian society

India	The chance to standardize undergraduate cariology teaching in our country according to the UCCC is quite high. Reforms in undergraduate and postgraduate curricula are considered	Domain I is quite vast and needs to be more structured to serve as the base for foundation for cariology

Russia	This would require even more efforts and time. The dental faculty of our university is in close connection with other faculties in the country. Therefore, a good chance exists for a collaboration for a discussion and standardization along the UCCC	Diagnostic criteria (early diagnosis instead of traditional diagnosis of caries at the stage of cavitation) Clinical decision making and minimal invasive approach in caries treatment decision In the short term, these modifications can be added to the elective part of the university program. In the long term, they should be implemented in the standardized Russian educational common program (main part)
